# Aquaculture and *mcr* Colistin Resistance Determinants

**DOI:** 10.1128/mBio.01229-17

**Published:** 2017-10-03

**Authors:** Felipe C. Cabello, Alexandra Tomova, Larisa Ivanova, Henry P. Godfrey

**Affiliations:** a Department of Microbiology and Immunology, New York Medical College, Valhalla, New York, USA; b Institute of Physiology, Faculty of Medicine, Comenius University in Bratislava, Bratislava, Slovakia; c Department of Pathology, New York Medical College, Valhalla, New York, USA; Indiana University Bloomington

**Keywords:** antimicrobial resistance, aquaculture, microbial ecology

## LETTER

We read with great interest the article in *mBio* regarding the discovery of a third colistin resistance determinant, *mcr-3*, in plasmid pWJ1 isolated from porcine *Escherichia coli* in China (1). This discovery and the subsequently reported discovery of a fourth colistin resistance determinant, *mcr-4* (2), provide further support for our hypothesis that the *mcr* determinants (phosphoethanolamine transferases) may have primarily or simultaneously originated in aquatic environments as a result of aquacultural activities, including integrated aquaculture, that bring together aquatic and terrestrial bacteria (3).

The over 45 million metric tons of fish, crustaceans, and mollusks produced by aquaculture in China in 2014 far surpass the nearly 15 million metric tons of these animals captured by Chinese fisheries (4). More than 50% of this aquacultural production is exported, and this fast-growing industry employs millions of people (4). We have previously suggested that some of the original events that generated plasmid-mediated colistin resistance genes *mcr-1* and *mcr-2* may have been stimulated by the heavy use of colistin and other antimicrobials in this industry in China through its facilitation of the capture and dissemination of potential colistin resistance genes from aquatic bacteria (3). Consistent with our hypothesis, *mcr-3* found in *Escherichia coli* encodes a protein that shows significant amino acid sequence identity with phosphoethanolamine transferases of *Aeromonas salmonicida* (84%), *Aeromonas hydrophila* (76%), and *Aeromonas piscicola* (77%), while *mcr-4* found in *Salmonella* encodes a phosphoethanolamine transferase with a 99% amino acid sequence identity with *Shewanella frigidimarina* (2). The *mcr-3*-containing plasmid pWJ1 has a type 1 integron, and it and the *mcr-4*-harboring bacteria also contain other antimicrobial resistance determinants [*floR*, *aac(6′)-Ib-cr*, *sul*, *aadA*, and *tetA*], which both we and others have found to be present in aquatic bacteria related to aquaculture (5), lending further credence to our hypothesis.

*Aeromonas* and *Shewanella* are pathogens of aquacultured fish and, like other fish pathogens, can be naturally resistant to colistin (see Table S1 in reference 1). That members of these genera are also human pathogens may be one of the elements that aids dissemination of resistance determinants originating in aquatic organisms to terrestrial pathogens through horizontal gene transfer steps that remain uncharacterized. While the shuttling of *mcr-3* and *mcr-4* determinants from aquatic bacteria to human pathogens appears to be carried out by plasmids, a conjugative plasmid of the IncHI2 compatibility group in the case of *mcr-3* (1) and a potentially mobilizable and transformable plasmid of the ColE type in the case of *mcr-4* (2), the relevant question is how *mcr* determinants carried on the chromosome in *Aeromonas* and *Shewanella* become plasmid bound and transferable. As we have previously suggested, recombination between chromosomal and plasmid DNA segments could be mediated by insertion sequences, IS*CR* sequences, transposons, bacteriophages, and the insertion and excision of plasmids into the chromosome (3, 6). Their dissemination among pigs could be achieved by infections of pigs with bacteria containing them under conditions of highly integrated terrestrial and aquatic husbandry and by horizontal gene transfer in the environment and the pig microbiome (6, 7).

We believe the increasing commercial availability on world markets of products of Chinese and other countries’ aquaculture might be also a factor in the rapid globalization of colistin-resistant bacteria and colistin resistance genes (1, 3, 6). Aquacultural activities could thus provide reactors for generating and disseminating new antimicrobial resistances and mechanisms into both aquatic and terrestrial environments (6). Even though antimicrobial use in aquaculture has been little acknowledged until recently, it clearly needs to be better assessed and regulated, and proximity to fish farms may well be considered a risk factor for acquisition of bacteria with *mcr* resistance determinants (6, 7).

## References

[1] Yin W, Li H, Shen Y, Liu Z, Wang S, Shen Z, Zhang R, Walsh TR, Shen J, Wang Y 2017 Novel plasmid-mediated colistin resistance gene *mcr-3* in *Escherichia coli*. mBio 8:e00543-17. doi:10.1128/mBio.00543-17. 28655818 PMC5487729

[2] Carattoli A, Villa L, Feudi C, Curcio L, Orsini S, Luppi A, Pezzotti G, Magistrali CF 2017 Novel plasmid-mediated colistin resistance *mcr-4* gene in *Salmonella* and *Escherichia coli*, Italy 2013, Spain and Belgium, 2015 to 2016. Euro Surveill 22:30589. doi:10.2807/1560-7917.ES.2017.22.31.30589. 28797329 PMC5553062

[3] Cabello FC, Godfrey HP 2017 Comment on: transferable resistance to colistin: a new but old threat. J Antimicrob Chemother 72:636–637. doi:10.1093/jac/dkw432. 27733518

[4] Food and Agriculture Organization 2016 The state of world fisheries and aquaculture. Contributing to food security and nutrition for all. Food and Agriculture Organization, Rome, Italy http://www.fao.org/3/a-i5555e.pdf.

[5] Tomova A, Ivanova L, Buschmann AH, Rioseco ML, Kalsi RK, Godfrey HP, Cabello FC 2015 Antimicrobial resistance genes in marine bacteria and human uropathogenic *Escherichia coli* from a region of intensive aquaculture. Environ Microbiol Rep 7:803–809. doi:10.1111/1758-2229.12327. 26259681

[6] Cabello FC, Godfrey HP, Buschmann AH, Dölz HJ 2017 Aquaculture as yet another environmental gateway to the development and globalisation of antimicrobial resistance. Lancet Infect Dis 16:e127–e133. doi:10.1016/S1473-3099(16)00100-6. 27083976

[7] Wang Y, Tian GB, Zhang R, Shen Y, Tyrrell JM, Huang X, Zhou H, Lei L, Li HY, Doi Y, Fang Y, Ren H, Zhong LL, Shen Z, Zeng KJ, Wang S, Liu JH, Wu C, Walsh TR, Shen J 2017 Prevalence, risk factors, outcomes, and molecular epidemiology of mcr-1-positive Enterobacteriaceae in patients and healthy adults from China: an epidemiological and clinical study. Lancet Infect Dis 17:390–399. doi:10.1016/S1473-3099(16)30527-8. 28139431

